# Miasis intestinal humana por *Eristalis tenax* en un niño de la zona urbana del municipio de Policarpa, Nariño, Colombia

**DOI:** 10.7705/biomedica.5400

**Published:** 2020-12-09

**Authors:** Álvaro Francisco Dulce-Villarreal, Angélica María Rojas-Bárcenas, José Danilo Jojoa-Ríos, José Fernando Gómez-Urrego

**Affiliations:** 1 Salud Ambiental, lnstituto Departamental de Salud de Nariño, Pasto, Colombia lnstituto Departamental de Salud de Nariño Pasto Colombia; 2 Dirección de Vigilancia y Análisis del Riesgo, Instituto Nacional de Salud, Bogotá, D.C., Colombia Instituto Nacional de Salud BogotáD.C Colombia; 3 Grupo de Investigación en Pediatría (GRINPED), Universidad Libre, Cali, Colombia Universidad Libre Universidad Libre Cali Colombia

**Keywords:** miasis, parásitos, enfermedades parasitarias, diarrea infantil, enfermedades gastrointestinales, larva, Myiasis, parasites, parasitic diseases, diarrea, infantile, gastrointestinal diseases, larva

## Abstract

La miasis se produce por la infestación con larvas de especies de moscas que afectan los tejidos u órganos de los vertebrados, incluido el ser humano. Puede clasificarse por caracterización entomológica o según el tropismo de las larvas en los tejidos. La miasis intestinal es poco frecuente y de difícil diagnóstico dada su sintomatología inespecífica, por lo que la única forma de confirmar el caso es mediante la identificación de las larvas expulsadas. Se presenta el primer caso reportado en el departamento de Nariño (Colombia) de miasis intestinal en un niño de seis años residente en la zona urbana del municipio de Policarpa, proveniente de una familia de nivel socioeconómico bajo, sin acceso a un adecuado tratamiento y sin disposición de aguas residuales y con insuficientes condiciones de saneamiento básico. El caso clínico se asoció con diarrea crónica, dolor abdominal y prurito anal, con la posterior expulsión de una larva cuyas características morfológicas correspondían a las de la mosca *Eristalis tenax.*

## Abstract (en)

Myiasis is a pathology caused by the infestation of fly larvae species which affects vertebrates' tissues or organs including humans. It can be classified entomologically or according to tissue tropism. Intestinal myiasis is rare and difficult to diagnose given its non-specific symptoms; the only way to confirm a case is by identifying the expelled larvae. This is the first case of intestinal myiasis reported in the department of Nariño (Colombia) in a 6-year-old child from a low-income family in the urban area of the municipality of Policarpa where there is no adequate treatment and no disposition of wastewater and basic sanitation conditions are insufficient. The clinical case was related to chronic diarrhea, abdominal pain, and anal pruritus with the subsequent expulsion of a larva identified by its morphological characteristics as *Eristalis tenax* fly larva.

La miasis es la infestación de tejidos u órganos (principalmente de ojos, piel, nariz, senos paranasales, garganta e intestino), vivos o muertos, por larvas de moscas ^1^. En los humanos se presenta principalmente en personas enfermas, de países en desarrollo y condiciones de saneamiento básico insuficientes ^2^^,^^3^. La miasis humana puede tener un curso benigno y asintomático o, por el contrario, producir graves problemas de salud e, incluso, la muerte ^4^.

Entomológicamente, la miasis puede originarse por parásitos obligados cuyas larvas requieren tejidos vivos para desarrollarse; por parásitos facultativos que se encuentran en tejidos en descomposición, vegetales y, a veces, en tejidos vivos, y por parásitos accidentales que se adquieren al ingerir alimentos contaminados y que ocasionan infestación ^5^^,^^6^.

*Eristalis tenax* es una especie de díptero que se encuentra en todas las regiones biogeográficas y es especialmente prevalente en las proximidades de los centros de actividad humana ^7^. En cuanto a su ciclo de vida, los huevos eclosionan y producen larvas que se alimentan de materia orgánica y se desarrollan durante dos a tres semanas. Las larvas poseen una formación respiratoria posterior a modo de tubo delgado retráctil similar a una cola, rasgo que facilita su identificación y explica que se las conozca como "larvas cola de ratón'.' Al llegar a los dos o tres centímetros, las larvas forman pupas que se depositan en la tierra durante ocho a 20 días hasta que eclosionan y aparecen las moscas adultas con aspecto de abeja melífera. Estas moscas son atraídas por el olor a putrefacción y desovan en lugares con materia orgánica en descomposición, por lo que sus huevos o larvas suelen encontrarse en sitios insalubres, sobre heridas infectadas o en vegetales en descomposición ^8^.

Se presenta el primer caso reportado en Nariño de miasis intestinal por *E. tenax* en un niño de seis años.

## Caso clínico

**Figura 1:**
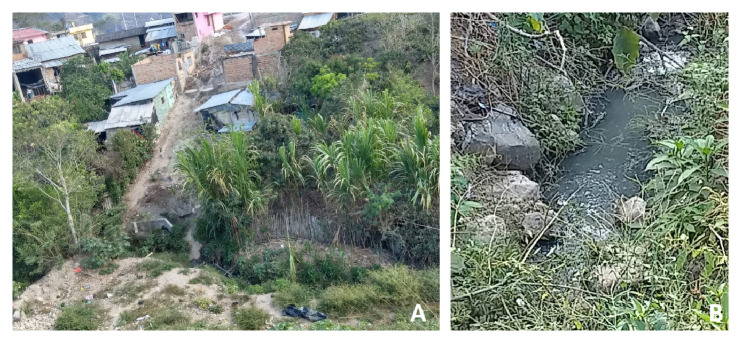
A. Barrio de residencia del paciente en el municipio de Policarpa, Nariño, Colombia. Nótese la cercanía de las casas a la cañada por donde fluyen las aguas residuales. B. Aspecto de la quebrada que recibe las aguas residuales

Se trata de un niño de seis años de edad, habitante de la zona urbana del municipio de Policarpa, Nariño, sur de Colombia, en un sector de estrato socioeconómico bajo con deficientes condiciones de saneamiento básico y sin canalización de aguas residuales, por lo que estas fluyen hacia el fondo de una cañada situada a pocos metros del barrio de residencia del menor (figura 1).

Desde los 18 meses de edad, el paciente venía presentando un cuadro clínico recurrente e intermitente de dolor abdominal de tipo cólico de intensidad moderada que remitía sin tratamiento, y se asociaba con enfermedad diarreica crónica no disentérica y prurito anal. En múltiples ocasiones fue llevado al puesto de salud local en donde se le trató con antiparasitarios convencionales y se le dieron recomendaciones de modificación de la dieta, a pesar de lo cual continuó con la misma sintomatología.

**Figura 2:**
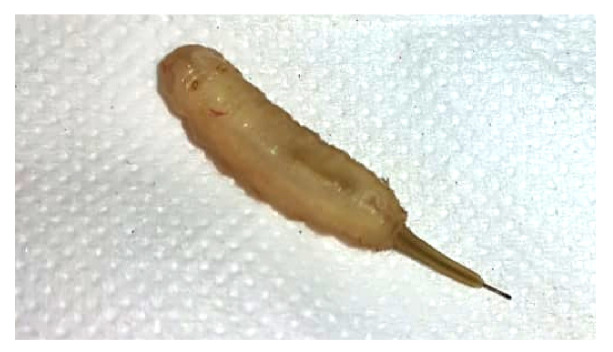
Larva de la mosca *Eristalis tenax* (larvas cola de rata) identificada en las heces del paciente. Nótese el cuerpo cilíndrico de color amarillo pardo y, en la parte posterior, una prolongación en forma de tubo delgado retráctil y móvil. Estas características ayudan a su identificación.

En una ocasión, mientras el paciente defecaba, la madre observó la expulsión por el recto de una larva de aproximadamente tres cm de longitud, cilíndrica, de color amarillo pardo, y rematada por una prolongación a modo de tubo delgado retráctil similar a una cola, que le servía de ayuda para moverse (figura 2). La madre recogió al animal en un recipiente y acudió de inmediato al puesto de salud local con el paciente. Allí nuevamente se le formuló un antiparasitario y se lo remitió para ser valorado en un establecimiento de salud con un nivel de atención superior, donde hicieron el examen coproscópico mediante el cual se detectó y se caracterizó el parásito como una larva de la mosca *E. tenax* (larva cola de rata). El paciente recibió tratamiento con nitazoxanida y albendazol y su familia se instruyó sobre las medidas de prevención de las infecciones gastrointestinales: lavado de manos, adecuada manipulación de alimentos y potabilización del agua para el consumo.

### Consideraciones éticas

Se siguieron las normas éticas para la investigación en seres humanos contenidas en la Resolución 008430 de 1993 del Ministerio de Salud de Colombia y se mantuvo la confidencialidad de la información del paciente.

## Discusión

La miasis humana es una enfermedad prevalente en los países tropicales que comúnmente afecta a habitantes de las áreas rurales y se asocia con condiciones insalubres de vida, sin agua potable ni adecuado tratamiento o canalización de aguas residuales, con las consecuentes deficiencias en la higiene de las personas. La materia orgánica en descomposición y el olor a putrefacción atraen a las moscas, las cuales depositan allí sus huevos ^2^^,^^9^^,^^10^. Estas condiciones de insalubridad en el entorno del paciente explican que se haya infectado con larvas de *E. tenax* y haya desarrollado miasis intestinal. El foco de infestación reconocido fueron las aguas residuales contaminadas de una quebrada en las inmediaciones de su domicilio.

Las miasis viscerales son causadas por la contaminación de los alimentos o el agua con huevos de moscas que son ingeridos de manera accidental. Se han descrito casos de miasis en el sistema digestivo ^1^^,^^8^^,^^11^^,^^12^, pero también en otras localizaciones poco usuales, como la tráquea y los pulmones ^13^^-^^15^, la nasofaringe ^15^^,^^16^, las vías urinarias ^17^ y los genitales ^18^^,^^19^.

La larva de *E. tenax* tiene forma alargada, sus movimientos son giratorios, su abdomen es cilíndrico, su longitud es de 3 cm y su diámetro de 0,5 cm, y termina en una larga cola retráctil de 4 a 5 cm ^10^^,^^12^, características que permitieron identificar esta especie como la causante de la miasis intestinal en el presente caso.

La miasis intestinal se puede manifestar con síntomas inespecíficos de dolor abdominal de tipo cólico intermitente y crónico, diarrea crónica, náuseas, y emesis; si se presenta en el recto o cerca al ano, puede haber sensación de masa y prurito; puede, además, cursar con sangrados gastrointestinales ^4^^,^^8^^,^^11^^,^^12^^,^^20^. El diagnostico final se logra mediante la identificación morfológica de las larvas expulsadas.

El tratamiento de la miasis intestinal incluye las soluciones isotónicas conjuntamente con laxantes para eliminar las larvas, así como medicamentos antiparasitarios ^8^^,^^12^^,^^21^. Además, son importantes las recomendaciones generales para la prevención de infecciones gastrointestinales, como un adecuado lavado de manos, la apropiada manipulación de los alimentos y la potabilización del agua de consumo.

## Conclusión

Las larvas de *E. tenax* pueden causar miasis intestinal, condición cuya sintomatologia es inespecífica (dolor abdominal y diarrea crónicos, náuseas, emesis, prurito anal), por lo que es necesario identificar las larvas expulsadas para diagnosticarla. Los lugares propensos a la presencia de miasis intestinal por *E. tenax* se caracterizan por ser insalubres, no contar con agua potable y tener un inadecuado tratamiento de las aguas residuales.
